# Attenuation of virulence in an apicomplexan hemoparasite results in reduced genome diversity at the population level

**DOI:** 10.1186/1471-2164-12-410

**Published:** 2011-08-12

**Authors:** Audrey OT Lau, Ananth Kalyanaraman, Ignacio Echaide, Guy H Palmer, Russell Bock, Monica J Pedroni, Meenakshi Rameshkumar, Mariano B Ferreira, Taryn I Fletcher, Terry F McElwain

**Affiliations:** 1 Programs in Genomics and Vector-borne Diseases, Department of Veterinary Microbiology & Pathology and Paul G. Allen School for Global Animal Health, College of Veterinary Medicine, Washington State University, Pullman, WA 99164-7040, USA; 2 School of Electrical Engineering and Computer Science, Washington State University, Pullman WA 99164-2752, USA; 3 Instituto Nacional Tecnologia Agropecuaria, 2300 Rafaela, Santa Fe, Argentina; 4 Tick Fever Centre, Biosecurity, Department of Primary Industries and Fisheries, 280 Grindle Road, Wacol, Qld. 4076, Australia; 5 Department of Medicine, Imperial College London, South Kensington Campus, London SW7 2AZ, UK

## Abstract

**Background:**

Virulence acquisition and loss is a dynamic adaptation of pathogens to thrive in changing milieus. We investigated the mechanisms of virulence loss at the whole genome level using *Babesia bovis *as a model apicomplexan in which genetically related attenuated parasites can be reliably derived from virulent parental strains in the natural host. We expected virulence loss to be accompanied by consistent changes at the gene level, and that such changes would be shared among attenuated parasites of diverse geographic and genetic background.

**Results:**

Surprisingly, while single nucleotide polymorphisms in 14 genes distinguished all attenuated parasites from their virulent parental strains, all non-synonymous changes resulted in no deleterious amino acid modification that could consistently be associated with attenuation (or virulence) in this hemoparasite. Interestingly, however, attenuation significantly reduced the overall population's genome diversity with 81% of base pairs shared among attenuated strains, compared to only 60% of base pairs common among virulent parental parasites. There were significantly fewer genes that were unique to their geographical origins among the attenuated parasites, resulting in a simplified population structure among the attenuated strains.

**Conclusions:**

This simplified structure includes reduced diversity of the variant erythrocyte surface 1 (*ves*) multigene family repertoire among attenuated parasites when compared to virulent parental strains, possibly suggesting that overall variance in large protein families such as Variant Erythrocyte Surface Antigens has a critical role in expression of the virulence phenotype. In addition, the results suggest that virulence (or attenuation) mechanisms may not be shared among all populations of parasites at the gene level, but instead may reflect expansion or contraction of the population structure in response to shifting milieus.

## Background

Pathogens adapt to maintain selective advantage in their environments. As few environments are themselves stable, pathogen adaptation is a dynamic and continuous process. This principle applies to virulence in which acquisition and loss of virulence is dynamic within a pathogen population, varying with host genetics, host immune status at the individual and population levels, and transmission efficiency. These shifts can be achieved through sexual reproduction where novel recombinations often lead to genome diversity [1]. However, for many multi-stage pathogens, this cannot occur during haploid life stages, and thus, they depend on mutations at the genome level or adopt phenotypic variation as a result of non-mutational mechanisms. Mutations associated with virulence (or attenuation) are frequently documented in eubacteria [2-4] and viruses [5,6]. However, there is a significant gap in our knowledge of virulence-associated gene mutations in protozoa, including pathogens which have a major impact on global public and animal health. With increased emphasis on development and delivery of attenuated vaccines for hemoparasites such as *Babesia, Theileria*, and *Plasmodium *spp. [7-9], the ability to predictably and stably attenuate these pathogens would be a significant step toward vaccine implementation.

In this study, we investigated a natural host-apicomplexan pathogen interaction, infection of cattle with *Babesia bovis*, to investigate virulence loss at the genome level using multiple strains of diverse geographical origin and genetic background. Virulent *B. bovis *infection results in a clinical syndrome characterized by severe hemolytic anemia, hyperthermia, and a syndrome of neurovirulence clinically and pathophysiologically similar to cerebral malaria [10]. However, through serial *in vivo *passages of virulent *B. bovis *in a splenectomized host, the virulence phenotype is gradually lost, and an attenuated *B. bovis *derivative is obtained. Animals infected with the attenuated derivative do not exhibit neurovirulence, do not require treatment, and are protected upon virulent parental challenge [11]. Reliable derivation of attenuated parasites is the basis for production of *B. bovis *live vaccine in *Babesia *endemic countries such as Australia, Israel and Argentina [9,12,13]. Furthermore, attenuated vaccine generated from strains isolated in Australia confer cross protection in cattle in Africa, Latin America and Southeast Asia [11,13], suggesting that immunogens are shared between geographically divergent strains.

In this report, we took advantage of the babesial attenuation procedure to compare coding regions across the complete genomes of distinct *B. bovis *strain pairs with distinguishable virulence and attenuation phenotypes in the natural host. With one virulent *B. bovis *genome sequence already published [14], we subsequently sequenced its attenuated derivative and two additional geographically distinct strain pairs using pyrosequencing technology. The results of a comprehensive genome analysis among all three virulent/attenuated strain pairs (six strains) are presented and discussed in the context of dynamic virulence acquisition and loss.

## Methods

Three *B. bovis *virulent and attenuated strain pairs were used in this study. T2Bo_vir is a strain previously isolated in Texas, USA and its genome sequence has been published [14]. T2Bo_att is the attenuated derivative of T2Bo_vir. L17_vir is a virulent field isolate that originated from an infected animal in Salta province, Northwest Argentina. L17_att is the attenuated derivative of L17_vir. T_vir, also referred to as E61, is a virulent isolate that originated from an infected animal in North Queensland, Australia. T_att, also referred to as F100, is the attenuated derivative of T_vir. For comparative analysis, the fully sequenced T2Bo_vir is used as the reference and all the strains used are designated as "test" strains.

### Attenuation of virulent *B. bovis *strains

Attenuation of the T strain (T_att) was achieved through 28 *in vivo *rapid passages from its original virulent parent, T_vir as previous described [11]. The subsequent attenuated T strain, T_att, was used as a vaccine in Australia between 1991 and 1993 [15].

The L17_att and T2Bo_att strains were also attenuated by rapid passage as described [11] and were evaluated after 24 and 29 *in vivo *passages, respectively. Before the attenuation procedure, T2Bo_vir and L17_vir strains were passed through non-infected *Rhipicephalus microplus *ticks to obtain stabilates. Liquid nitrogen preserved stabilates of L17_vir or T2Bo_vir were used to inoculate the first splenectomized calves with 1.2 × 10^8 ^parasitized erythrocytes after which the infected blood was collected in heparin (5-10 IU/ml) five days post-infection and transferred intravenously to a second splenectomized calf. Subsequent passages were identical with blood collected and transferred with an average of 5 ± 1 day interval.

### Evaluation of attenuated derivatives

Evaluation of the T_att strain has been described [15]. To assess the attenuation of L17_att and T2Bo_att *B. bovis *strains, 16-month old Holstein steers were subcutaneously inoculated with 10^7 ^parasitized erythrocytes of each strain. Briefly, age-matched steers raised in tick-free areas and sero-negative for *B. bovis *were inoculated with each putatively attenuated strain. The average normal body temperature and hematocrit of each steer were established during three consecutive days immediately before the initiation of the experiment. Daily assessment of the same parameters, as well as parasitemia was carried out between days 6 and 18 after inoculation. Steers were treated any time that hyperthermia > 41.0°C was observed during three consecutive days, hematocrit was < 15%, parasitemia > 0.5%, and/or neurological signs were observed. All animals infected with virulent *B. bovis *strains (T2Bo and L17) required treatment while those infected with the attenuated derivatives did not (Table 1), confirming the successful attenuation of the virulent L17 and T2Bo strains.

**Table 1:** *In vivo *evaluation of *Babesia bovis *attenuation

Inoculated strain	T2Bo_vir	T2Bo_att	L17_vir	L17_att
# of animals	12	18	12	18
Average age of animal (mth)	16.5	15.5	17	16
# of animals requiring treatment*	12/12	0/18	12/12	0/18

* treatment criteria include one of the following. Hyperthermia > 41°C for 3 consecutive days, percent parasitemia > 0.5%, hematocrit < 15% and neurological signs such as ataxia, excess salivation, head pressing, lateral head torsion and recumbency w/. fully extended head. Vir, virulent phenotype; att, attenuated phenotype; T2Bo, Tex strain; L17, Argentina strain.

### Short term *in vitro *propagation of *B. bovis *and genomic DNA extraction

T2Bo_vir or _att and L17_vir or _att strains from splenectomized calves were used to establish short term continuous *in vitro *propagation (< 30 days of culture) as described previously [16,17]. Parasitized erythrocytes of each strain were frozen in liquid nitrogen, cryopreserved with 10% polyvinylpyrolidone (PVP) and stored in liquid nitrogen until use.

T_vir was made from pooled blood from 10 animals from a property called 'Reedy Beds' Tharingowa via Townsville in far North Queensland. This Australian virulent strain was passaged twice in splenectomized calves to generate enough parasitized erythrocytes for gDNA extraction. Short term *in vitro *culturing was not performed for this strain.

Genomic DNA of *B. bovis *infected erythrocytes was obtained as previously described [18]. Southern dot blot hybridization was carried on test strain gDNA to ensure that all samples contained < 10% bovine host gDNA contamination (data not shown) prior to pyrosequencing.

### Pyrosequencing

The *B. bovis *gDNA for T2Bo_vir was completely sequenced by a classical method (Sanger shotgun) and contains two small assembly gaps and one physical gap [14]. *B. bovis *gDNA for the remaining five strains was submitted to the 454 pyrosequencing facility (http://www.454.com) for shotgun sequencing (see Additional file 1, table s1). Briefly, genomic DNA was randomly fragmented into small 300 to 800 bp fragments. Adapters were ligated to the generated fragments, thus creating a library of DNA fragments. These fragments were immobilized onto DNA capture beads and individually sequenced on a PicoTiterPlate device. The average read lengths of T_att, T_vir, L17_att, L17_vir and T2Bo_att were 101 bp, 251 bp, 215 bp, 250 bp and 386 bp, respectively. The generated sequences were then assembled into a number of unordered and unoriented contigs using the GS De Novo Assembler Software resulting in a consensus sequence. For more information on pyrosequencing and the assembly tool, please visit http://www.454.com/applications.

### Comparative genomic analyses

Comparative genomics analysis was performed on all six strains using the T2Bo_vir strain as the reference strain and the remaining strains as test strains. *Blastn *[19] was used to assign contigs to chromosome scaffolds (chromosomes 1-4, apicoplastic, mitochondrial) based on the topmost hits. Ordering and orientation of the contigs in the draft assemblies was achieved by mapping along the T2Bo_vir reference genome. Scaffolded contigs were visualized for manual inspection using the Mauve software package [20,21].

Inter-strain genome comparisons involving two or more genomes from either within or across the virulent and attenuated groups were carried out using the Mauve tool. These genome alignments also served to visualize and quantify the overall quality of the individual draft assemblies for the test strains. A combination of Unix bash and Perl scripts were written to compute genome- and gene-wide coverage of the individual draft assemblies relative to the reference, and to calculate various other measures that relate to the distribution of assembly gaps and the impact of repetitive content (e.g., *ves *genes) on assembly. These scripts were written specifically for these genomes and will be made freely available upon request.

### SNP identification and gene analysis

**Figure 1:**
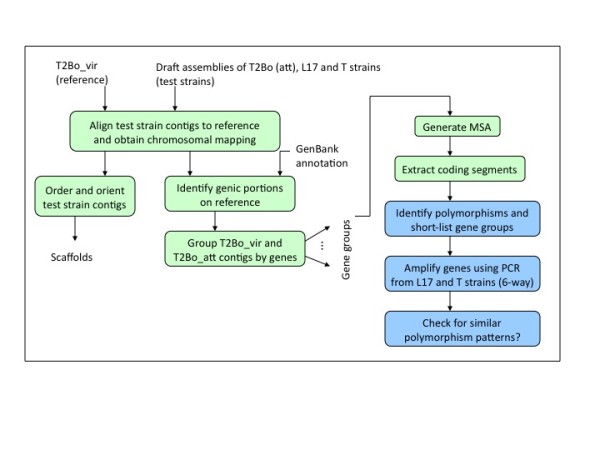
**Flowchart for comparative gen-centric (genic) analysis**. Green boxes indicate computational steps and blue boxes denote steps that require manual intervention.

Consistent polymorphisms within the gene segments of the chromosomes that differentiate the parental virulent from the derived attenuated strains in all three regional pairs were identified. Available sequence analysis programs were used whenever possible, and custom scripts and programs were implemented to automate the pipeline. The steps of the analysis are as follows (also see Figure 1).

Initially, gene boundaries were marked along the T2Bo_vir contigs using available GenBank annotation. Subsequently, the subset of T2Bo_att contigs that mapped to the genic portions of T2Bo_vir were identified and isolated. The coordinates for this mapping information were determined using MUMmer program [22]. This subset was further sub-divided into gene groups (≤ e value of 10^-5 ^as an arbitrary cutoff for gene determination), where contigs mapping to the same gene were binned together. Long contigs mapping to multiple genes, although from different parts, were made as part of each of the corresponding gene groups. After binning, a multiple sequence alignment (MSA) was generated separately for each group using the CAP3 fragment assembly tool [23]. Note that using an MSA would restrict the similarity detection process to full-length sequence at the nucleotide level. This highly sensitive strategy was used deliberately as a means to flag the slightest of differences between the two forms of T2Bo. Automated scripts were written to trim the MSAs of their leading and trailing gaps, following which intronic portions were excised, retaining only the coding segments. Output of MSAs was then manually examined by visual inspection using an alignment program (MacVector, vers.11.1). Two types of polymorphic events were identified during the search process - base substitutions such as single nucleotide polymorphisms (SNPs) and sequence gaps. The latter event could have arisen due to true insertions/deletions (indels) or simply lack of sequence data. A list of candidate genes resulting from the two-way (T2Bo_vir and _att only) comparison was generated for further evaluations. MSA of these genes were subsequently generated for the remaining two strain pairs. Genes that were commonly found to be different between all strain pairs were then selected for re-sequencing.

Separately, SNPs and indels in the different test strains relative to the reference were identified by comparing each of the test strains against the reference using the MUMmer tool and its default parameters and a customized-written script (freely available for download upon request).

### Re-sequencing and analysis

Gene specific primers were generated and synthesized (http://idtdna.com) for re-sequencing of the final candidate genes. PCR amplicons of genomic DNA from T2Bo virulent and attenuated strains were used in the comparison to confirm SNPs or indels that were identified *in silico *between this strain pair. If polymorphisms were detected from this comparison, genomic DNA from L17 and T strain pairs were then used for re-sequencing to determine if the observed polymorphisms were also found in the other strains; thereby making the process into a 6-way comparison. High fidelity Taq (Invitrogen) was used in all re-sequencing to minimize PCR-generated errors. Amplicons were cloned into pCR4.1TOPO (Invitrogen) and multiple clones per PCR were sequenced (WSU Sequencing Core Facility). Sequence analysis was performed using MacVector vers.11.1. All putative protein-coding sequences were included in the analysis except for *ves1 *gene family (n = 119), BBOV_I002740 and BBOV_IV029790 which encode for variant erythrocyte surface antigens, a 200 kDa antigen and a hypothetical protein, respectively. Repetitive sequences within these genes prohibited accurate alignments for comparison.

## Results

### Pyrosequencing achieved high genomic coverage of all strains

Pyrosequencing was utilized to sequence the genomes of the test strains. Resulting contigs for each test strain were partitioned based on the sequences (gene groups) from the reference T2Bo_vir strain. This process resulted in a total of 3,671 gene groups in the test strains. The total number of contigs (by chromosome) was the highest for T_vir while T2Bo_att had the lowest number of contigs (Additional file 1, table s2). Overall, the assemblies corresponding to the attenuated strains were of a better quality than the virulent strains of L17 and T, with longer contigs and N50 contig lengths (see Table 2). Genomic and gene coverages of all the five draft assemblies over the T2Bo_vir reference were computed (Table 3). The genomic coverages were high: 93% for T2Bo_att, 90% for L17_vir and _att, 84% for T_vir and 89% for T_att. Importantly, the coverages of the draft assemblies on the gene portion compared to the T2Bo_vir reference were also equally high: of the approximately 4.2 Mbp (52%) of the T2Bo_vir genome that are coding sequences, the contigs of the test strains covered 85% to 88% in the T strains, 90% in the L17 strains and to 93% in the T2Bo_att strain.

**Table 2:** Assembly statistics of the five *Babesia bovis *genomes in comparison to the reference T2Bo_vir

	**T2Bo_Vir**.	**T2Bo_Att**.	**L17_Vir**.	**L17_Att**.	**T_Vir**.	**T_Att**.
Number of contigs	14	1,397	6,520	1,168	9,449	4,049
N50 contig length (bp)	1,797,577	113,852	5,452	87,856	4,772	43,696
Longest contig (bp)	2,593,320	366,332	358,771	288,976	64,333	298,977
Total contig length (bp)	8,146,795	8,155,482	9,099,385	7,892,637	9,387,722	8,020,682

T2Bo, *Babesia bovis *Texas strain; L17, *B. bovis *Argentinian strain; T, *B. bovis *Australian strain; vir, virulent; att, attenuated.

**Table 3:** Genomic and genic coverages of the five *Babesia bovis *draft assemblies over the reference T2Bo_vir

A. Genome-wide coverage			
Strain	% base coverage by contigs	Total gap length (bp)	Number of gaps
T2Bo_att	93.33%	545,969	277
L17_vir	90.59%	769,743	1,095
L17_att	90.53%	774,280	480
T_vir	84.45%	1,271,819	1,765
T_att	89.26%	878,389	803
**B. Genic coverage**			
**Strain**	**% base coverage by contigs**	**Gap length in genes (bp)**	

T2Bo_att	93.00%	299,288	
L17_vir	89.79%	436,406	
L17_att	89.60%	444,495	
T_vir	84.63%	656,517	
T_att	88.37%	496,798	

T2Bo, *Babesia bovis *Texas strain; L17, *B. bovis *Argentinian strain; T, *B. bovis *Australian strain; att, attenuated; vir, virulent.

Individual chromosomal coverages were also calculated. Chromosome 4, the largest of all the chromosomes (2.59 Mbp), had the highest genomic coverage among nuclear chromosomes, with each test strain covering on average 94% of the reference strain. In contrast, chromosome 1, the smallest of the nuclear chromosomes (1.19 Mbp), had the lowest coverage with an average of 80% (data not shown) among the test strains. Gene coverage of each test strain by chromosome and the two organelles was also investigated (Table 4). Similar coverage scenarios were obtained with the highest gene coverage for chromosome 4 and lowest for chromosome 1. There was 100% coverage of the mitochondrial genomes for all strains while ~92% coverage of the apicoplast genomes was achieved (Table 4). The overall gene coverage of the T strains (Australian strains) is the lowest while T2Bo_att is the highest. It is important to note that the positioning of the contigs in the test strains was achieved by mapping them along the reference strain, as paired-end sequencing was not done. Therefore, there is not sufficient information in the assembly to fully reconstruct or detect the presence of re-arrangement/recombination events between these strains.

**Table 4:** Genic coverage by chromosome of the five *Babesia bovis *strains over the reference T2Bo_vir

	T2Bo_att	L17_att	T_att	L17_vir	T_vir	(Average % coverage)
Chr. 1	82.22%	79.17%	77.82%	77.98%	74.22%	78.28%
Chr. 2	92.74%	89.73%	86.85%	89.34%	84.14%	88.56%
Chr. 3	94.80%	90.63%	89.44%	91.23%	84.33%	90.01%
Chr. 4	96.30%	93.23%	93.13%	94.06%	89.94%	93.33%
Mitochondria	100.00%	100.00%	100.00%	100.00%	96.93%	99.39%
Apicoplast	93.91%	91.04%	91.97%	90.70%	90.60%	91.64%

T2Bo, *Babesia bovis *Texas strain; L17, *B. bovis *Argentinian strain; T, *B. bovis *Australian strain; att, attenuated; vir, virulent.

### Sequence divergence decreases with attenuation

**Figure 2:**
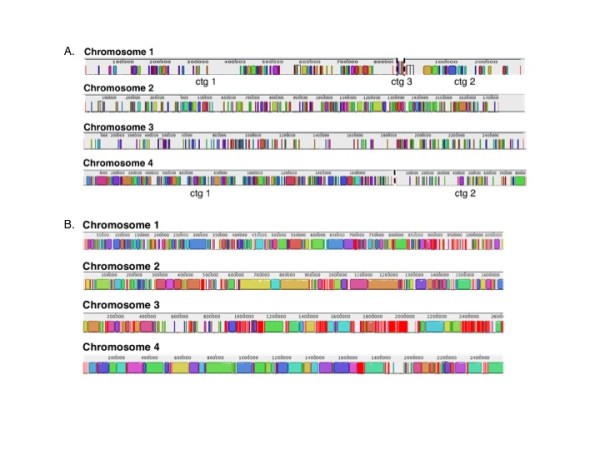
**Global chromosomal consensus alignments between all virulent (A) and attenuated (B) strains of T2Bo, L17 and T virulent**. Chromosomes are not drawn to scale. Color blocks represents individual assembled contigs. Gaps on chromosomes represent sequences that are missing in at least one of the strains in the corresponding group. Smaller contigs (ctg) on chromosome 1 (n = 5) are omitted for the virulent strain alignments.

**Figure 3:**
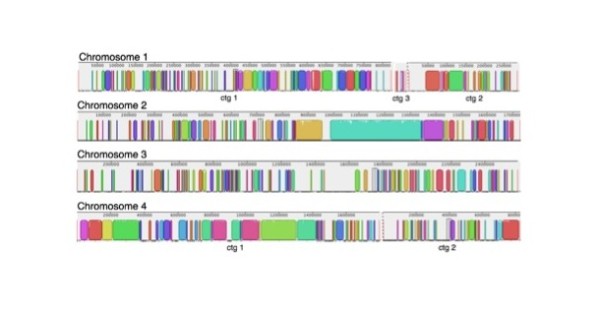
**Chromosomal alignments between the reference virulent strain, T2Bo_vir and L17_vir**. Chromosomes are not drawn to scale. Color blocks represents individual assembled contigs. Gaps on chromosomes represent sequences that are missing in at least one of the strains in the corresponding group. Smaller contigs (ctg) on chromosome 1 (n = 5) are omitted for the virulent strain alignments.

**Figure 4:**
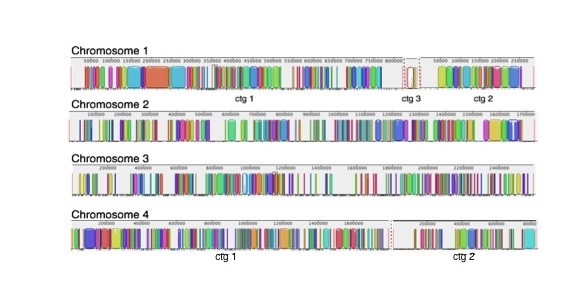
**Chromosomal alignments between reference virulent strain, T2Bo_vir and T_vir**. Chromosomes are not drawn to scale. Color blocks represents individual assembled contigs. Gaps on chromosomes represent sequences that are missing in at least one of the strains in the corresponding group. Smaller contigs (ctg) on chromosome 1 (n = 5) are omitted for the virulent strain alignments.

**Figure 5:**
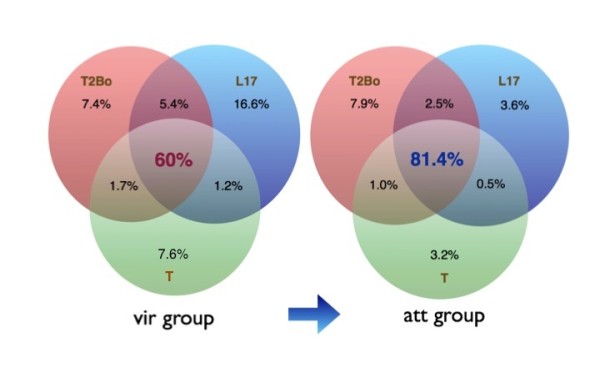
**Whole genome comparison between all three strains**. Pink, blue and green regions are strain-specific sequences of T2Bo, L17 and T strains, respectively. Overlapping area in the center of each Venn diagram represents nucleotide sequences (coding and non-coding) that are shared among all virulent or attenuated strains. Areas shaded in pink, blue and green are strain-specific nucleotide sequences found in T2Bo, L17 and T strains, respectively. Both Venn diagrams are not drawn to scale.

Chromosomal alignments of all virulent or attenuated strains were plotted to investigate the degree of similarity within each group (virulent vs. attenuated). Figure 2 illustrates the consensus alignments of the virulent and attenuated strains on each chromosome. Chromosome 4 and 1 are the most conserved and divergent chromosomes of all *B. bovis *virulent strains at the nucleotide level, respectively (Figure 2A). Each gap represents genomic regions that are missing in at least one of the strains, while the colored blocks denote conserved sections that are present in all virulent strains. In contrast to the virulent strains, the attenuated strains contain fewer gaps in each of the four chromosomes, suggesting a higher degree of sequence conservation among all the attenuated strains (Figure 2B). As observed in the virulent strain comparison, sequences on chromosome 4 are the most conserved among attenuated strains while the larger gap lengths in chromosome 1 illustrate the potential diversity of this chromosome among the three attenuated *B. bovis *strains. Figures 3 and 4 are individual virulent genome alignments by chromosome with T2Bo_vir reference, illustrating that the gaps as a result to missing sequences are different in locations for both L17_vir and T_vir as compared with the reference strain. Notably, the overall similarity at the nucleotide level (coding and non-coding) among the attenuated strains was observed to be considerably higher (81%) than among the virulent strains (60%) (Figure 5). This analysis also illustrates that the number of strain-specific ("unique") sequences decreases with attenuation.

**Figure 6:**
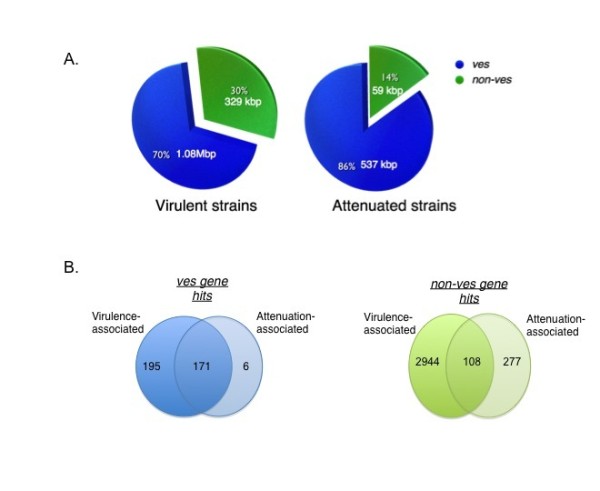
**Distribution of strain-specific sequences between virulent and attenuated groups**. Strain-specific sequences can be classified into *ves *(blue) and non-*ves *(green) gene families and (B) total number of strain-specific *ves *and non-*ves *gene hits in the virulent and attenuated groups.

**Figure 7:**
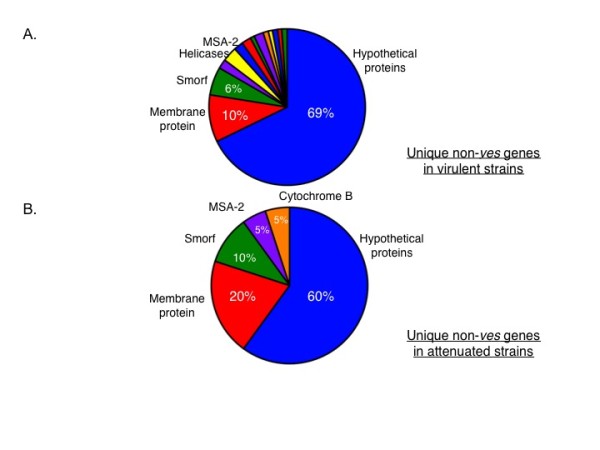
**Functional assignments of strain-specific non-*ves *genes in the virulent (A) and attenuated (B) populations**. Colored sections within each pie represent different gene families and only those > 5% of the total are labeled.

To further investigate the contents of the unique portions of these genomes, genomic contig sequences specific to each strain among the three virulent (or three attenuated) group of genomes were first identified. Such sequences were labeled as "strain-specific" and are shown to occupy the pink, blue and green regions of the Venn diagrams (Figure 5). The number of bases covered by strain-specific sequences (genic and non-genic) is decreased by 35% in the virulent group when compared to the attenuated group (3.64 Mbp to 1.28 Mbp). In order to identify genes that are represented within the strain-specific contigs of the genomes, these sequences were blasted (using *blastx*) against the NCBI GenBank NR database and alignment hits that spanned more than 100 amino acids and an e-value score ≤ 0.05, were reported. This filtering process reduced the total strain-specific contig sizes to 1.41 Mbp in the virulent group, and 597 Kbp in the attenuated group (data not shown). The strain-specific coding sequences were further partitioned into *ves *and non-*ves *genes. Interestingly, 70% of virulent strain-specific gene bases were *ves*-associated (Figure 6). The remaining non-*ves *strain-specific sequences encoded mostly genes for hypothetical genes (> 60%) and for, but not limited to, translation and membrane proteins (Figure 7). Among all the virulent strain-specific *ves *genes (n = 372), 45% are retained in the attenuated strains, which translates into 97% of the strain-specific *ves *in the attenuated strains that are retained from the virulent population (Figure 6B). Only 3% of all strain-specific *ves *genes are found exclusively in attenuated strains (Figure 6B). In comparison to 45% of virulent strain-specific *ves *that are retained in the attenuated population, only 3% of strain-specific non-*ves *genes from the virulent group are carried over to the attenuated strains (Figure 6B). Energy production-related genes are among those present in the attenuated strain-specific non-*ves *genes that are not detected in the virulent group (Figure 7). The *ves*: non-*ves *ratio of roughly 2:1 for the virulent group changed with attenuation to approximately 6:1, consistent with great reduction of non-*ves *diversity generated through attenuation.

**Figure 8:**
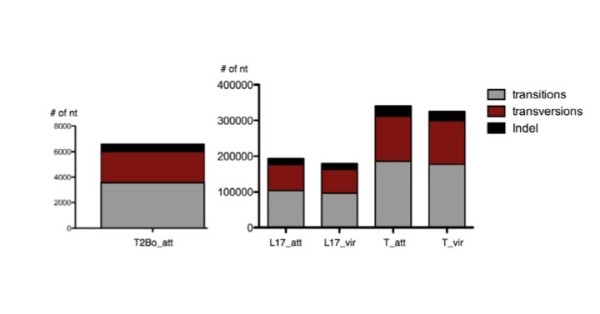
**Distribution of transversions (crimson), transitions (grey), insertions and deletions (Indel) (black) in the five *Babesia bovis *genomes as compared to the reference strain**.

**Figure 9:**
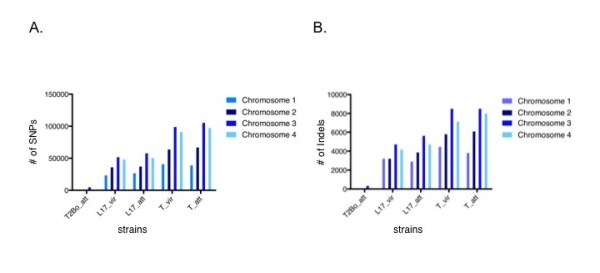
**Total number of single nucleotide polymorphisms (SNPs) (A) and insertions and deletions (Indel) and (B) by chromosome of all test strains in comparison to the reference, T2Bo_vir**.

Single nucleotide polymorphism (SNP) analysis was conducted between the reference, T2Bo_vir strain and each of the five test strains at the genome level. As shown in Figure 8, transitions (purine to purine) were more commonly detected than transversions (purine to pyrimidine). Within the transversion subgroups, both T strains have a higher number of transversions than the T2Bo_att and L17 strain pairs. A similar pattern was observed when insertion and deletion (indel) analysis was performed (Figure 9). While it was observed that chromosome 3 consistently contains a greater number of SNPs and indels than chromosomes 1, 2 and 4 in all the test strains (Figure 7) when these numbers were normalized to the number of genes in each chromosome or the size of the chromosomes, the disproportionate higher incidence of SNPs and indels observed on chromosome 3 is no longer present (data not shown).

### No common gene differences are shared among all virulent or attenuated strain pairs

In order to identify common gene differences between all virulent and all attenuated strains, MSA was performed initially between T2Bo_vir reference and T2Bo_att to generate a list of genes with either nucleotide differences or simply the absence of any MSA output between this parent-offspring pair. A total of 226 genes were identified for further investigation (data not shown). Members of this gene list were used to generate MSA between L17_vir and L17_att and T_vir and T_att. A second gene list comprising gene differences shared among the three strain pairs resulted (Additional file 1, table s3). Out of these 14 genes, half of these have annotational information while the remaining genes encode for hypothetical proteins. However, re-sequencing of all 14 genes showed that although common polymorphisms were found between all attenuated/virulent strain pairs, these polymorphisms were insignificant in the translated products using SIFT [24].

## Discussion

The ability to predictably attenuate *B. bovis *by multiple passages through splenectomized cattle has been used in *Babesia *endemic regions to produce the only available vaccine that can protect animals from severe clinical disease [11,25]. The attenuated parasites are infectious but do not cause clinical disease requiring treatment. A similar loss of virulence has been reported in related organisms such as *Theileria, Toxoplasma *and *Plasmodium *[26-28]. We took advantage of this attenuation model system to perform whole genome comparison of three different *B. bovis *virulent parent and attenuated daughter strain pairs. Our initial focus was to identify gene changes shared among all attenuated strains in an effort to determine if there was a common pathway to attenuation.

Pyrosequencing technology [29] enabled us to achieve high genome coverage for all the *B. bovis *test strains (n = 5), with the highest coverage for T2Bo_att, followed by L17 and T strains (Table 3). The quality of the assembly varied from strain to strain. Most notably the N50 contig length was considerably less for the virulent strains (L17_vir and T_vir) than their attenuated counterparts (L17_att and T_att) (Table 2), indicating that the assembly for the virulent forms of these two strains was more fragmented despite the higher depth used in sequencing the virulent parasites (see Additional file 1, table s1). This result is most likely a manifestation of the high level of sequence diversity among the virulent population - i.e., reads emerging from a diverse population are less likely to collapse into long contigs during assembly. Despite the difficulties in assembly encountered in virulent strains, the study was not negatively impacted since genomic coverage of these strains was still substantially high (> 84%) when aligned over the T2Bo_vir reference genome.

Comparison of shared sequences between the three strain pairs showed that there are more shared sequences between the North and South American strains (L17 and T2Bo) (Figure 5). This suggests that L17 and T2Bo are genetically more closely related to each other than to the Australian T strain. More SNPs were identified in both T strains when the orthologues were compared to those in L17 and T2Bo. Many of these SNPs, nonetheless, were synonymous changes. However, this observation suggests that the codon usage is similar between L17 and T2Bo. The greater level of T strain sequence diversity compared to the reference strain may contribute to increased difficulty in recognizing orthologues to the reference genes using our stringent gene identification criteria, subsequently resulting in more and larger sequence gaps.

When genomic- and genic-wide coverages by chromosome (Table 3) in all strains were computed, irrespective of the quality of the coverage, chromosomes 1 and 4 consistently had the poorest and best coverage, respectively. A noticeable difference between these two chromosomes is the distribution of variant erythrocyte surface 1 (*ves*) gene family members. The *ves *gene family is the largest gene family in the *B. bovis *(T2Bo strain) genome and contains multiple repeat sequences [10,14]. Chromosome 1 has the highest number of known *ves*, with at least 42 of the 119 total *ves *in the genome, representing ~16% of the total gene content of chromosome 1. By contrast, only ~3% of the total gene content of chromosome 4 is comprised of *ves*. Since pyrosequencing is not typically suited to repetitive regions [30], this could explain the lower gene coverage observed in the assembly of chromosome 1 when compared to chromosome 4 (Table 4). To validate this hypothesis, we mapped all the *ves *locations in the two chromosomes onto the contigs and investigated if they were located within the gap regions. First, we observed that a high percentage (> 84%) of the *ves *gene bases fall into assembly gaps consistently across all chromosomes, reflecting the high complexity inherent in sequencing the *ves *gene family. Next, we compared the distribution of the assembly gaps to *ves *and non-*ves *regions in chromosome 1 and 4. Analysis showed that 46% of the gaps in chromosome 1 are attributable to *ves *genes while the remaining 54% include non-*ves *genes, intergenic and intronic regions. In contrast, only 21% of the gaps in chromosome 4 are attributable to *ves *(data not shown). These data support our conclusion that the poor coverage of chromosome 1 in the virulent strains as compared to chromosome 4 is due to the clustering of *ves*.

The total number of strain-specific sequences in each virulent strain is greater than that of the attenuated strains (Figure 5), explaining in part why there are more gaps in the consensus chromosomal alignments among the virulent strains (Figure 2A). Approximately 60% of the sequences are shared among all virulent strains and 81% among the attenuated strains. This is not a simple artifact of comparing sequences obtained by different techniques, as comparison of the L17_vir and T_vir strains, both of which were pyrosequenced, resulted in the same significant gap differences when compared to the attenuated strains. This reduced diversity in strain-specific sequences may be attributed to a change in population structure during the attenuation process, resulting in a simplified or more uniform attenuated population. Evidence of a similar phenomenon has been reported in *Theileria *and *Plasmodium *[28,31]. However, our study is the first demonstration of the change in population structure at the whole genome level. Among the strain-specific gene sequences, *ves *predominates in both the virulent and attenuated strains (Figure 6A). Interestingly, 97% of the total strain-specific attenuation-associated *ves *genes are also present in at least one virulent strain (Figure 6B), suggesting that the attenuation process did not select either or against a specific repertoire or for a unique repertoire of variant genes. Due to their polymorphic nature and repetitive sequences as well as a physical gap of approximately 150 kbp containing *ves *genes in chromosome 1 of the T2Bo_vir genome sequence, members of this gene family were not individually analyzed in our study. Nonetheless, results from the analysis of the strain-specific *ves *gene sequences between virulent and attenuated strains imply that there is likely considerable overlap of the known *ves *repertoire between the virulent parent and its attenuated derivative. This may account for the ability of the attenuated parasites to protect against clinical disease caused by virulent strains after only one infection cycle. In contrast, multiple clinical infections are typically required to generate clinical immune protection against *P. falciparum *infection [32,33], possibly reflecting the more diverse repertoire of variant genes such as *var *(encoding PfEMP1) among virulent isolates [34].

Analysis of non-*ves1 *strain-specific gene sequences between virulent and attenuated strains also showed a significant decrease in the number of strain-specific sequences as a result of attenuation. Approximately 60% of these strain-specific non-*ves *sequences are of unknown function while the remainder in both virulent and attenuated groups includes membrane proteins, merozoite surface antigen-2 and small open reading frames (smorfs) (Figure 7). Non-*ves *strain-specific sequences in virulent strains also include genes not found in the attenuated non-*ves *unique sequences group. This suggests that some genes uniquely present in the virulent strains are selected against during the attenuation process, while others are enriched.

There were few non-synonymous differences between coding sequences in all three strain pairs which might account for the shared phenotypic characteristics of virulence or attenuation as all non-synonymous changes resulted in tolerated translated products. This may imply that virulence may be multigenic, involving more than one gene in different strains and depending on the strain, may involve different genes, all belonging to the same pathway. Attenuation may also result from selection of a subpopulation whose virulence factors fail to reach a threshold expression level for virulence, such a mechanism has been reported by Colinet *et al*. [35] for *Leptopilina boulardi*, a parasitic wasp of *Drosophila melanogaster*.

Our comparative data indicate that *ves *gene members are repeatedly the dominant strain-specific sequences for each strain. *Ves *is 70% and 86% of the total strain-specific sequences in the virulent and attenuated groups, respectively. As attenuation was achieved, however, the number of the strain-specific *ves *sequence decreased. An attractive hypothesis based on the observation of population-based *ves *gene complexity is that virulence is associated with a unique repertoire of VESA proteins expressed on the infected erythrocyte surface. This is consistent with the mechanism used to generate attenuation. Rapid passage of parasites in cattle without a spleen may result in selection of a non-cytoadherent population that would normally be cleared in spleen intact animals, while cytoadherent parasitized erythrocytes sequester in tissue capillaries. Thus, the small percentage loss of certain *ves *genes may be responsible for the phenotypic difference between the virulent and attenuated strain pairs. However, an equally plausible hypothesis of attenuation mechanism can be due to the reduction of strain-specific non-*ves *genes. The reduction of approximately half of all strain-specific non-*ves *genes post-attenuation is remarkable. Lastly, phenotypic changes exhibited in the infected host may be controlled at the transcriptional or protein level. While non-synonymous polymorphisms were predicted to result in tolerated changes, synonymous single nucleotide polymorphisms differentiating virulent and attenuated parasites in all three strain pairs were also found among the 14 genes. It is plausible that synonymous nucleotide changes may play a role in virulence phenotype, although the mechanism of such is currently unknown.

## Conclusions

In summary, this study surveyed and compared genomes of three genetically related *B. bovis *strain pairs of diverse geographical background and with distinct differences in the ability to cause severe clinical disease (virulence). Common virulence factor(s) at the gene level were not consistently found among all virulent *B. bovis *strains or their attenuated derivatives. However, the virulent parasite population gene pool was significantly more complex than in attenuated *B. bovis *parasites. Gene complexity was a result of diversity in both a multi-copy variant gene family and multiple single copy genes. Thus, while there were no common genes consistently associated with attenuation or virulence in these related strain pairs, the distinct and reproducible phenotype of the attenuated derivatives in infected animals may be a result of contraction of virulence-associated genes of varying function during the passage of parasites in the natural host. Virulence mechanisms may also be associated with differences at the transcriptional or protein level, including the *ves *repertoire, or variations in intergenic or intronic regions [31]. For instance, Iyer *et al*. recently showed that increased expression of a rhoptry protein in a rodent malaria, *P. yoelii *(*py*235), resulted in the invasion of a wider range of erythrocytes and thus, greater virulence [36]. Alternatively, virulence may be achieved by various means such as epigenetic factors, all of which contribute to the overall phenotype exhibited in infected host [37,38].

The complete genomic sequences for the five test strains are available at http://www.eecs.wsu.edu/~ananth/WSU_B-bovis_RawData.

## Authors' contributions

All authors read and approved the final manuscript. AOTL contributed to the design, data analyses and drafting of the manuscript while AK contributed to data analyses and drafting of the manuscript. MJP contributed to the generation of the re-sequencing data. MK contributed to the analysis of the *in silico *data. IE and MBF were responsible for the generation and validation of two virulent and attenuated strain pairs (L17 and T2Bo). RB and TIF generated and validated the virulent and attenuated T strain pair. GHP and TFM contributed to the design of the experiments and preparation of the manuscript.

## Supplementary Material

Additional file 1 **Table s1 Sequencing related information for the *Babesia bovis *strains**. Additional sequencing information showing the coverages of the six *B. bovis *genomes. Each genome coverage is calculated relative to the size of the T2Bo_vir genome as the reference, which is 8.3 Mbp. Table s2. Contig distribution by chromosome of the three *Babesia bovis *strain pairs. This table illustrates the total number of assembled contigs of each genome and their chromosomal distributions of the six *B. bovis *strains. Table s3. List of candidate genes for re-sequencing. Candidate genes that required re-sequencing after comparative gene analysis among all six *B. bovis *strains was completed. Click here for file
