# Supplementary material for: Attenuation of virulence in an apicomplexan hemoparasite results in reduced genome diversity at the population level
Source: BMC Genomics. 2011 Aug 12;12:410. doi: 10.1186/1471-2164-12-410 (PMC3166950; doi:10.1186/1471-2164-12-410)
Supplement: Additional file 1 — Table s1 Sequencing related information for the Babesia bovis strains. Additional sequencing information showing the coverages of the six B. bovis genomes. Each genome coverage is calculated relative to the size of the T2Bo_vir genome as the reference, which is 8.3 Mbp. Table s2. Contig distribution by chromosome of the three Babesia bovis strain pairs. This table illustrates the total number of assembled contigs of each genome and their chromosomal distributions of the six B. bovis strains. Table s3. List of candidate genes for re-sequencing. Candidate genes that required re-sequencing after comparative gene analysis among all six B. bovis strains was completed. [file 1471-2164-12-410-S1.DOC]

Table s1. Sequencing related information for the *Babesia bovis* strains. Coverage is calculated relative to the size of the T2Bo_vir genome as the reference, which is 8.3 Mbp

|  | T2Bo_vir. | T2Bo_att. | L17_vir. | L17_att. | T_vir. | T_att. |
| --- | --- | --- | --- | --- | --- | --- |
| Technology | Sanger | 454 | 454 | 454 | 454 | 454 |
| Coverage of genome sequence | 8.8x | 28x | 25x | 16x | 33x | 21x |

T2Bo, *Babesia bovis* Texas strain; L17, *B. bovis* Argentinian strain; T, *B. bovis* Australian strain; att, attenuated; vir, virulent.

Table s2. Contig distribution by chromosome of the three *Babesia bovis* strain pairs.

|  | T2Bo_vir. | T2Bo_att. | L17_vir. | L17_att. | T_vir. | T_att. |
| --- | --- | --- | --- | --- | --- | --- |
| Chr. 1 | 7 | 177 | 1,088 | 220 | 1,572 | 946 |
| Chr. 2 | 1 | 144 | 1,265 | 317 | 2,076 | 750 |
| Chr. 3 | 1 | 867 | 2,571 | 316 | 3,310 | 1,512 |
| Chr. 4 | 3 | 200 | 1,585 | 307 | 2,468 | 813 |
| Apicoplast | 1 | 5 | 8 | 5 | 15 | 13 |
| Mitochondria | 1 | 2 | 2 | 2 | 6 | 5 |

T2Bo, *Babesia bovis* Texas strain; L17, *B. bovis* Argentinian strain; T, *B. bovis* Australian strain; att, attenuated; vir, virulent.

Table s3. Candidate genes that required re-sequencing after comparative gene analysis among all six *Babesia bovis* strains.

| Gene identification | SNP positions | Annotation |
| --- | --- | --- |
| BBOV_I001300 | **G402A** | Membrane protein |
| BBOV_I002740 | **A542G** | 200kDa antigen |
| BBOV_II000740 | A616T; **T956G** | Spherical body protein2 |
| BBOV_II002880 | C897T | 85kDa protein |
| BBOV_III005620 | T138C; **A184G** | Hypothetical protein |
| BBOV_III006890 | A272G; T276C;  C3569G | Conserved hypothetical protein |
| BBOV_III006910 | T780G | Conserved hypothetical protein |
| BBOV_III007070 | **A658C**; **A676G**;  **A724C** | Hypothetical protein |
| BBOV_III007090 | T265C | Proteasome A- and B-type family protein |
| BBOV_III007130 | **G55C**; **G320C** | HesB-like domain containing protein |
| BBOV_III010020 | **A17C**; **G20T** | Histone acetyltransferase, ELP3 family protein |
| BBOV_III010720 | T190G | Hypothetical protein |
| BBOV_III010750 | **T758G**; **T775C**;  A899G | Hypothetical protein |
| BBOV_IV002180 | C233G; C571T;  T661A | Hypothetical protein |

**Bold letters**, non-synonymous SNPs
